# Association of Survival and Immune-Related Adverse Events With Anti-PD-1/PD-L1 and Anti-CTLA-4 Inhibitors, Alone or Their Combination for the Treatment of Cancer: A Systematic Review and Meta-Analysis of 13 Clinical Trials

**DOI:** 10.3389/fonc.2021.575457

**Published:** 2021-02-25

**Authors:** Leyin Zhang, Leitao Sun, Yiwen Zhou, Jieru Yu, Yingying Lin, Harpreet S. Wasan, Minhe Shen, Shanming Ruan

**Affiliations:** ^1^The First Clinical Medical College of Zhejiang Chinese Medical University, Hangzhou, China; ^2^Department of Medical Oncology, The First Affiliated Hospital of Zhejiang Chinese Medical University, Hangzhou, China; ^3^The Second Clinical Medical College of Zhejiang Chinese Medical University, Hangzhou, China; ^4^College of Basic Medical Science, Zhejiang Chinese Medical University, Hangzhou, China; ^5^Department of Cancer Medicine, Hammersmith Hospital, Imperial College Healthcare NHS Trust, London, United Kingdom

**Keywords:** immune checkpoint inhibitor, combination therapy, cancer, efficacy, immune-related adverse events, systematic review and meta-analysis

## Abstract

**Background:**

Cancer, with sustained high mortality, is a worldwide threat to public health. Despite the survival benefit over conventional therapies shown in immune checkpoint inhibitor (ICI), only a minority of patients benefit from single ICI. But combination therapy holds the promise of achieving better efficacy over monotherapy. We performed a systematic review and meta-analysis to assess the efficacy and safety of ICI-based combination therapy for cancer.

**Methods:**

A search was conducted to retrieve relevant studies in electronic databases and major conferences. Two investigators independently performed data extraction, making a systematic data extraction, assembly, analysis and interpretation to compare the overall survival (OS), progression-free survival (PFS), overall response rate (ORR), all and high grade immune related adverse events (IRAEs) between combination therapy and monotherapy. Therefore, only the studies satisfying the criteria were included. Finally, we performed subgroup, sensitivity, and publication bias analysis to examine the heterogeneity and bias of resources.

**Results:**

A total of 2,532 patients from thirteen studies were enrolled. Compared to ICI alone, combination therapy, with a high risk and high grade IRAEs for the majority of all, offers a better survival benefit (OS: HR: 0.86, 95% CI: 0.76 to 0.98; PFS: HR: 0.79, 95% CI: 0.69 to 0.90) and objective response (ORR: RR: 1.91, 95% CI: 1.40 to 2.60).

**Conclusions:**

ICI-based combination therapy was confirmed as the optimum treatment for cancer, especially when using specific dosage and regimen to treat certain tumor types with no absolute demand for the detection of PD-L1 expression. Meanwhile, attention should also be paid on potential toxicity, especially the IRAEs.

## Introduction

Cancer is a worldwide threat to public health and deprives over 9.56 million normal people of lives annually (1). Without surgery, chemotherapy, radiotherapy, or targeted therapy, immune checkpoint inhibitor (ICI) has shown remarkable efficacy as the treatment for multiple types of cancers (2), especially non-small cell lung cancer (3) and melanoma (4). So far, based on meta-analysis of controlled clinical trials, Food and Drug Administration (FDA) has approved six ICIs used for dozens of indications, including Avelumab, Ipilimumab, Nivolumab, Durvalumab, Pembrolizumab and Atezolizumab (5). Despite the improvement in overall survival and progression-free survival, the objective response of ICI monotherapy still remained low, with only 10 to 30 percent acquiring benefit from a single ICI. The majority of cancer patients failed to receive long-term benefits for the inevitable emergence of drug-resistance. However, researchers found that combination therapy is an effective option to tackle the problems after a great number of clinical trials, such as ICI monotherapy in combination with immunomodulator (6), chemotherapy (7), targeted therapy (8, 9), radiotherapy (10), vaccine (11), and others (12). Additionally, since the mechanisms of immune evasion involve multiple abnormal expressions of target points, ICI-based combination therapy could improve the therapeutic effect. It is believed that therapeutic prospect of ICI is turning from monotherapy to combination therapy, and a better efficacy might be achieved with ICI-based combination. Although ICI-based combination has brought significant hope to cancer therapeutics, as a result of toxic responses derived from the unbalanced activation of immune system, their clinical applications were also limited by immune-related adverse events (IRAEs) (13, 14).

Currently, while several meta-analyses have assessed the efficacy and safety of ICI-based combination therapy, most studies focus on the single type of cancer so that it is hard to evaluate the differences among multiple cancers. Besides, few studies explore the influence of potential subgroup differences, such as dosage, regimen and cancer type of ICIs (15–17). Moreover, in terms of safety of ICIs, in spite of the organ-specific IRAEs from toxic effect related to ICIs which has been discussed by published meta-analysis, they provided no detailed analysis on the differences between all-grade and high-grade adverse events (18, 19).

Therefore, we performed a systematic review and meta-analysis on 13 clinical trials using anti-PD-1/PD-L1 and anti-CTLA-4 inhibitors, alone or their combination, to comprehensively compare the efficacy and safety of ICI-based combination therapy with monotherapy in the treatment for patients with advanced and metastatic cancer. Moreover, we also attach great importance to the specific medication regimen, dosage of medicine, different types of cancer and others.

## Methods

Our team performed this meta-analysis according to the PRISMA statement guidelines (CRD42020183356) (20, 21), and had registered at International Prospective Register of Systematic Reviews for this work.

### Search Strategy

Aiming for exploring the efficacy and safety of ICI-based combination therapy and monotherapy, two investigators searched for relevant trials from three electronic databases including PubMed, Embase, Cochrane Library, and specific search strategies were shown in **Supplementary Method 1**. In addition, we also reviewed major meeting abstracts and presentations from ASCO, ESMO, and CSCO to identify the most updated studies, and clinical trial databases were also in the search. All trials were, without language limitation, related to the publication from initiation to April 12, 2020.

### Eligibility Criteria

Articles that meet all the following criteria are considered eligible for inclusion: (1) Prospective randomized trials in patients who were diagnosed with carcinoma; (2) ICI-based combination therapy is the treatment for participants in intervention group; (3) In control group, participants are treated with ICI-based monotherapy; (4) Studies have data available for OS and PFS as outcome measures. Meanwhile, an article would be excluded if it met one of the following cases: (1) Case report, review, meta-analysis, animal or *in vitro* research; (2) Only presented as abstracts without available full original text; (3) Trials compared ICI-based combination therapy to chemotherapy, radiotherapy, or targeted therapy. (4) Studies failed to provide data for survival analysis, such as OS, PFS, or survival curve. Two independent investigators only selected the literatures that fulfilled our predefined criteria after the first selection by viewing title and abstract of each article; then they skimmed through full texts to decide ultimate studies. All disagreements during the selection progress were discussed and resolved by all the investigators through consultation.

### Data Extraction

With PRISMA, two investigators individually extracted valid and complete data from full text of each included article. Firstly, we extracted information including first author, publication time, follow-up, trial design, phase, line of treatment, and total number, median age, gender of patient, drug administration in each group, and data available for hazard ratio (HR) and Relative Risk (RR) with 95% confidence interval (95% CI). Secondly, on comparison of the efficacy, the combination between HR and 95% CI for overall survival (OS) and Progression-Free Survival (PFS) was considered as the primary endpoint on the basis of intention to treat (ITT) analysis, which is derived from the initial treatment assignment but not the treatment eventually received. Thirdly, to ensure the safety, we deemed RR with 95%CI for immune related adverse events (IRAEs) as a secondary endpoint, which was calculated by the number of patients with IRAEs in each arm from all included trials. Moreover, the RR with 95%CI for Overall Response Rate (ORR) was also used as a secondary endpoint. Each clinical trial’s supplement was also in the search to assure that we exhaustively collected complete relevant data of each study with no omission.

### Bias Assessment

With the Cochrane Collaboration’s tool for the risk of bias (ROB) assessment, two independent investigators assessed ROB of the eligible studies and scored it as high, low, or unclear risk of bias. The evaluation contained the following characteristics covering selection bias (randomization sequence generation), selection bias (allocation concealment), performance bias (blinding of participants and personal), detection bias (blinding of outcome assessment), attrition bias (incomplete outcome data), reporting bias (selective reporting), and other biases. All investigators discussed and reached a consensus during bias assessment.

### Statistical Analysis

After previous steps, all available data were extracted from included trials. The primary endpoint of this meta-analysis was to compare OS and PFS between ICI-based combination therapy and monotherapy. The combination between HRs and 95% CI was used to express OS and PFS. The investigators calculated by Review Manager 5.3, and used STATA 15 to pool the data and produce the forest plots. HR <1 favored the intervention group (ICI-based combination therapy), whereas HR >1 favored the control group (ICI-based monotherapy). To analyze ORR and IRAEs, we calculated all the RRs and 95% CIs with data extracted from each trials, which based on the absolute numbers in patients assigned to ICI-based combination therapy compared with monotherapy and presented the objective response and all-grade, high-grade (≥3) IRAEs, respectively. RR <1 for ORR and IRAEs indicated a higher response rate and toxicity in the control group, while RR >1 is the opposite.

For statistical heterogeneity, we conducted the I² test, regarding a value greater than 25, 50, and 75% as an indicator of mild, moderate, and high heterogeneity, respectively. If P <0.10 or I^2^ >50%, the assumption of homogeneity was identified as invalid and a random-effects model was applied to pool effect size; if the opposite, data were calculated through a fixed-effects model. Furthermore, the investigators employed subgroup analysis and meta-regression analysis to deeply explore the heterogeneity and its potential influence.

As for cases that amount of studies reaching to ten or more, we performed funnel plots and visually assessed the asymmetry to explore possible publication biases. Besides, Egger’s test and Begg’s test were used to evaluate publication bias when the number of studies exceeded ten and twenty respectively. By estimating the average HRs in the absence of each study, the investigators performed the sensitivity analysis to test the robustness of each included trial to different aspects from methodological bias. A two-tailed p value of less than 0.05 meant statistically significance.

## Results

### Literature Search and Eligible Studies

**Figure 1:**
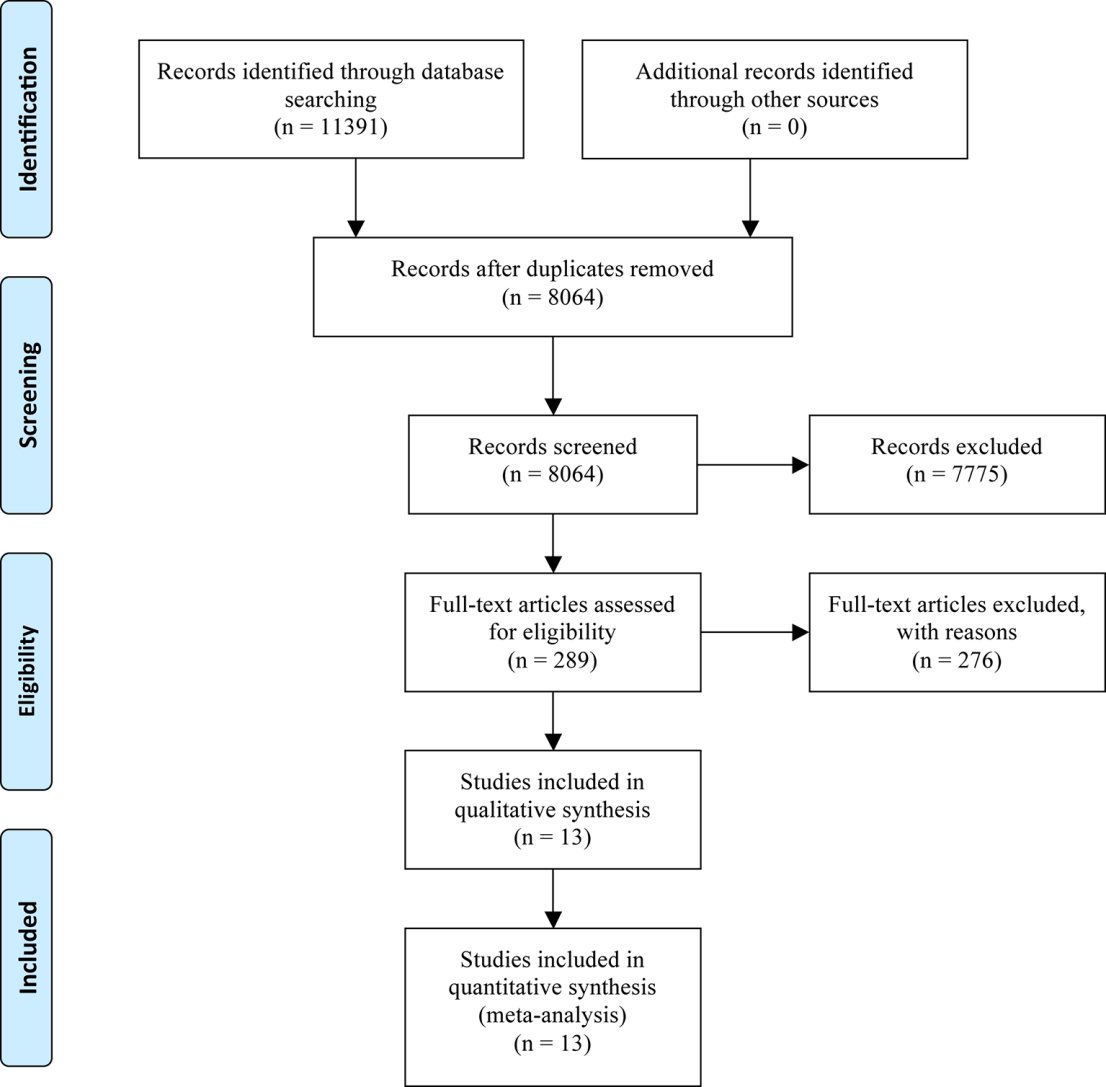
Flow diagram showing inclusion and exclusion of literature screening.

After excluding 3,327 duplicate articles of the initial search publications from online database and other sources, a total of 8,064 articles were retrieved in terms of the title and abstract, and 7,775 articles were removed in light of the following considerations and issues: review, case report, systematic reviews, and meta-analysis, only abstract without useful results, not randomized trials, not using ICI-based monotherapy in control group, not using ICI-based combination therapy in intervention group, targeted therapy, chemotherapy, radiotherapy, used in either group, and single-arm study design. Then we skimmed through the remaining 289 articles by full-text assessment and finally identified 13 RCTs. The specific search and selection steps are summarized in **Figure 1**.

### Characteristics of Included Trials and Patients

All trials were done in advanced or metastatic settings, among which three trials were performed in melanoma, two trials in small cell lung cancer, while the remaining were in other types of carcinomas. Seven trials were involved in phase II, four in phase I/II, and only one trial in phase I and III. Of the eligible studies, nine and two trials regarded Nivolumab and Ipilimumab as control regimen, respectively. ICI-based monotherapy was compared with Nivolumab (1 mg/kg) + Ipilimumab (3 mg/kg) in seven trials and with Nivolumab (3 mg/kg) + Ipilimumab (1 mg/kg) in six trials.

A total of 2,532 patients from thirteen studies, aged between 18 and 92, were included in our meta-analysis. Average follow-up for patients treated with ICI-based combination therapy was 6.5 to 54.6 months while for those treated with ICI-based monotherapy was 3.3 to 36.0 months. The major characteristics and results of included studies are shown in **Table 1** and **Supplementary Table 1**.

**Table 1:** Characteristics of included studies in this meta-analysis.

Author, year	Masking	Tumor type	Phase	No. of patients (n)	Male	Intervention arm			Control arm			HR (95% CI) for OS	HR (95% CI) for PFS
						Regimen	Dosage (mg/kg)	No. of patients (n)	Regimen	Dosage (mg/kg)	No. of patients (n)		
Antonia, 2016	Open-label	Small Cell Lung Cancer	I/II	213	128 (60.1%)	Nivolumab + Ipilimumab	1 + 3 Q3W	61	Nivolumab	3 Q2W	98	0.80 (0.56, 1.14)	0.72 (0.57, 0.91)
							3 + 1 Q3W	54				1.04 (0.73, 1.48)	0.95 (0.79, 1.16)
D’Angelo, 2018	Open-label	Sarcoma	II	85	41 (48.2%)	Nivolumab + Ipilimumab	3 + 1 Q3W	42	Nivolumab	3 Q2W	43	0.73 (0.44, 1.23)	0.71 (0.52, 0.98)
Hodi, 2016	Double-blind	Melanoma	II	142	95 (66.9%)	Nivolumab + Ipilimumab	1 + 3 Q3W	95	Ipilimumab	3 Q3W	47	0.74 (0.43, 1.26)	0.36 (0.22, 0.56)
Long, 2018	Open-label	Melanoma	II	60	48 (80.0%)	Nivolumab + Ipilimumab	1 + 3 Q3W	35	Nivolumab	3 Q2W	25	1.09 (0.51, 2.31)	0.43 (0.24, 0.79)
Omuro, 2018	Open-label	Glioblastoma	I	40	25 (62.5%)	Nivolumab + Ipilimumab	1 + 3 Q3W	10	Nivolumab	3 Q2W	10	1.33 (0.57, 3.10)	1.19 (0.79, 1.78)
							3 +1 Q3W	20				1.19 (0.55, 2.59)	0.98 (0.64, 1.48)
Scherpereel, 2019	Open-label	Pleural Mesothelioma	II	125	100 (80.0%)	Nivolumab + Ipilimumab	3 + 1 Q2W + Q6W	62	Nivolumab	3 Q2W	63	0.83 (0.50, 1.40)	0.80 (0.58, 1.11)
Yelena, 2018	Double-blind	Esophagogastric Cancer	I/II	160	124 (82.7%)	Nivolumab + Ipilimumab	1 + 3 Q3W	49	Nivolumab	3 Q2W	59	0.91 (0.61, 1.35)	0.76 (0.59, 1.00)
							3 + 1 Q3W	52				1.26 (0.88, 1.81)	0.86 (0.67, 1.10)
Larkin, 2019	Open-label	Melanoma	III	945	610 (64.6%)	Nivolumab + Ipilimumab	1 + 3 Q3W	314	Nivolumab	3 Q2W	316	0.91 (0.73, 1.14)	0.79 (0.66, 0.94)
									Ipilimumab	3 Q2W	315	0.61 (0.50, 0.75)	0.47 (0.40, 0.55)
Kelly, 2019	Open-label	Esophagogastric Cancer	Ib/II	88	63 (71.6%)	Durvalumab + Tremelimumab (2L)	20 + 1 Q4W	27	Durvalumab	10 Q2W	24	0.31 (0.16, 0.60)	0.87 (0.66, 1.16)
												0.62 (0.29, 1.32)	0.99 (0.65, 1.50)
						Durvalumab + Tremelimumab (3L)	20 + 1 Q4W	25	Tremelimumab	10 Q2W	12	2.13 (0.76, 6.00)	1.25 (0.34, 4.68)
												1.03 (0.62, 1.71)	1.01 (0.76, 1.36)
O’Reilly, 2019	Open-label	Pancreatic Ductal Adenocarcinoma	II	64	34 (52.0%)	Durvalumab + Tremelimumab	20 + 1 Q4W	32	Durvalumab	20 Q4W	32	1.03 (0.62, 1.71)	1.01 (0.76, 1.36)
Ready, 2020	Open-label	Small Cell Lung Cancer	I/II	243	147 (60.5%)	Nivolumab + Ipilimumab	1 + 3 Q3W	147	Nivolumab	3 Q2W	96	0.99 (0.75, 1.31)	0.89 (0.76, 1.03)
Siu, 2018	Open-label	Head and Neck Squamous Cell Carcinoma	II	267	220 (82.4%)	Durvalumab + Tremelimumab	20 + 1 Q4W	133	Durvalumab	10 Q2W	67	0.99 (0.69, 1.43)	1.13 (0.82, 1.56)
									Tremelimumab	10 Q4W	67	0.72 (0.51, 1.03)	0.73 (0.53, 1.01)
Zamarin, 2020	Open-label	Ovarian Cancer	II	100	NA	Nivolumab + Ipilimumab	3 + 1 Q3W	51	Nivolumab	3 Q2W	49	0.79 (0.44, 1.42)	0.53 (0.34, 0.82)

NA, not available.

### Assessment of Methodological Bias

The random sequence was generated by interactive voice or web response system only in six trials (22–27). In addition to (28–31) the rest of the studies succeeded to perform with adequate allocation concealment. Just three trials (32, 33) provided the detailed information about the blinding of participants and personnel, and all trials were given the blinding of outcome assessment. There was no obvious attrition bias existing in four trials (34). And all trials reported reliable and unselective results without reporting and other biases (**Supplementary Figures 1**, **2**).

### Efficacy

**Figure 2:**
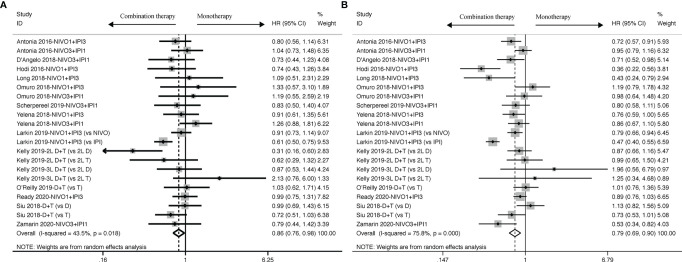
Forest plots of OS **(A)** and PFS **(B)** for cancer patients treated with ICI-based combination therapy *versus* monotherapy, presented as HR between two groups with a 95% CI.

The survival benefit was in favor of Nivolumab plus Ipilimumab combination therapy in terms of PFS (HR: 0.79, 95% CI: 0.69 to 0.90, **Figure 2B**) when compared to ICI-based monotherapy in control group, which revealed patients treated with ICI-based combination therapy received more survival benefits in reducing the risk of recurrence and progression. And, similar to PFS, it was proven by OS analysis that ICI-based combination therapy was also superior to Nivolumab or Ipilimumab alone (HR: 0.86, 95% CI: 0.76 to 0.98, **Figure 2A**). Besides, there were mild and high heterogeneity existing across all the included trials both in OS (I^2^ = 43.5%, *P = 0.018*) and PFS (I^2^ = 75.8%, *P < 0.001*) subsets, thus we ended up applying random-effects model to calculate the survival results.

### Selected Subgroup Analysis

**Figure 3:**
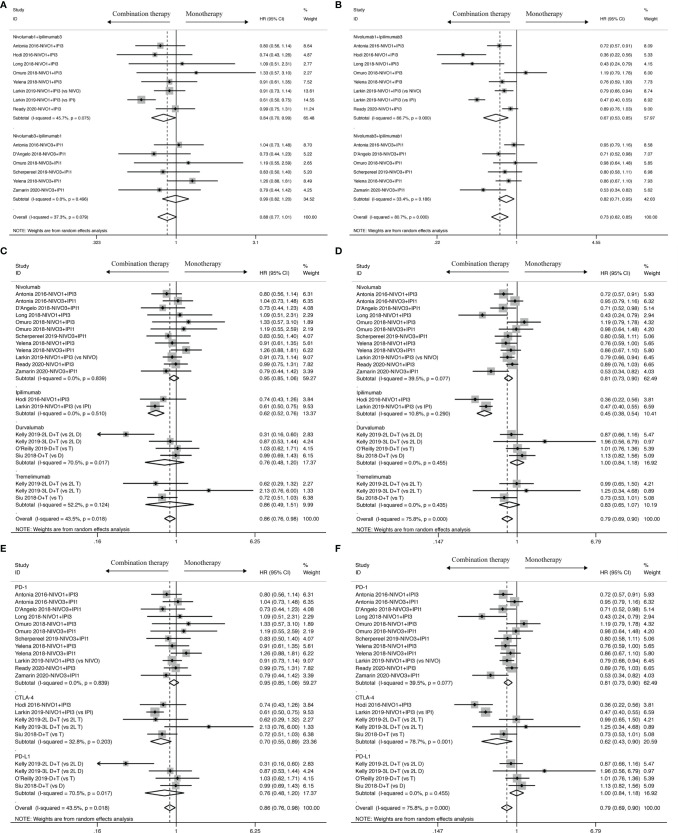
Subgroup analysis of HR and 95% CI of OS for cancer patients treated with ICI-based combination therapy *versus* monotherapy on the basis of specific dosage **(A)**, control drugs **(C)** and specific target of control drugs **(E)**; Subgroup analysis of HR and 95% CI of PFS for cancer patients treated with ICI-based combination therapy *versus* monotherapy on the basis of specific dosage **(B)**, control drugs **(D)** and specific target of control drugs **(F)**.

To figure out potential resources for heterogeneity, subgroup analysis was done in accordance to different dosages of ICIs in the intervention group, different regimens and its targets in the control group (**Figure 3**). PFS benefit was observed when compared to patients treated with Nivolumab or Ipilimumab in the control group (Nivolumab: HR: 0.81, 95% CI: 0.73 to 0.90; Ipilimumab: HR: 0.45, 95% CI: 0.38 to 0.54), but not with Durvalumab and Tremelimumab (Durvalumab: HR: 1.00, 95% CI: 0.84 to 1.18; Tremelimumab: HR: 0.83, 95% CI: 0.65 to 1.07). OS benefit, however, was only observed when compared with Ipilimumab in the control group (HR: 0.62, 95% CI: 0.52 to 0.76), but not with other control drugs (Nivolumab: HR: 0.95, 95% CI: 0.85 to 1.06; Durvalumab: 0.76, 95% CI: 0.48 to 1.20; Tremelimumab: HR: 0.86, 95% CI: 0.49 to 1.51).

In addition to the control group regimen, the specific molecular ICI targets also played a role in determining clinical effects. Cancer patients with combination therapy received a better OS benefit (HR: 0.70, 95% CI: 0.55 to 0.89) when compared to those with anti-CTLA-4 inhibitor in the control group, but failed to receive any benefit when compared to either anti-PD-1 inhibitor (HR: 0.95, 95% CI: 0.85 to 1.06) or anti-PD-L1 inhibitor (HR: 0.76, 95% CI: 0.48 to 1.20). But in PFS analysis, in addition to showing an advantage when compared to anti-CTLA-4 inhibitor (HR: 0.62, 95% CI: 0.43 to 0.90), we also found a PFS benefit only in favor of anti-PD-1 inhibitor (HR: 0.81, 95% CI: 0.73 to 0.90) rather than anti-PD-L1 inhibitor (HR: 1.00, 95% CI: 0.84 to 1.18).

Moreover, Nivolumab in combination with Ipilimumab achieved a better PFS benefit whatever the dosage was (Nivolumab1+ Ipilimumab3: HR: 0.67, 95% CI: 0.53 to 0.85; Nivolumab3 + Ipilimumab1: HR: 0.82, 95% CI: 0.71 to 0.95), along with a OS benefit in low-dosage Nivolumab plus high-dosage Ipilimumab (Nivolumab1 + Ipilimumab3: HR: 0.84, 95% CI: 0.70 to 0.99), but failed to provide any OS benefit in high-dosage Nivolumab plus low-dosage Ipilimumab (Nivolumab3 + Ipilimumab1: HR: 0.99, 95%CI: 0.82 to 1.20).

### PD-L1 Expression as Biomarker and Predictor

PD-L1 expression was divided into positive and negative by using a cut-off value (<1% and ≥1%) to explore the predictive effects of PD-L1 expression in the immune response of combination therapy.

**Figure 4:**
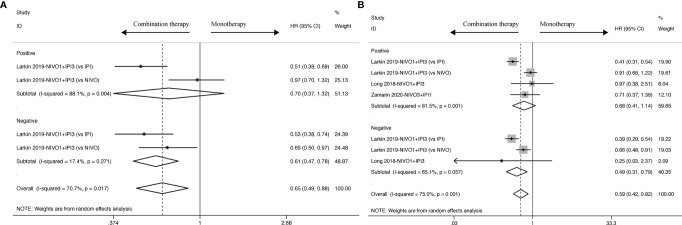
PD-L1 biomarker analysis of pooled HR and 95%CI of OS **(A)** and PFS **(B)** for cancer patients assigned to combination therapy, compared with those assigned to monotherapy.

OS benefit favored combination therapy in cancer patients with negative PD-L1 expression as shown in **Figure 4A** (HR: 0.61, 95% CI: 0.47 to 0.78), but not in those with positive PD-L1 expression (HR: 0.70, 95% CI: 0.37 to 1.32). As shown in OS analysis, cancer patients with negative PD-L1 expression also obtained a PFS improvement from combination therapy (HR: 0.49, 95% CI: 0.31 to 0.79) other than those with positive PD-L1 expression as shown in **Figure 4B** (HR: 0.68, 95% CI: 0.41 to 1.14). Moreover, ORR analysis was statistically significant for neither positive PD-L1 (RR: 1.78, 95% CI: 0.72 to 4.41) nor negative PD-L1 (RR: 0.45, 95% CI: 0.06 to 3.40) expression as shown in **Supplementary Figure 4**.

### Overall Response Rate

**Figure 5:**
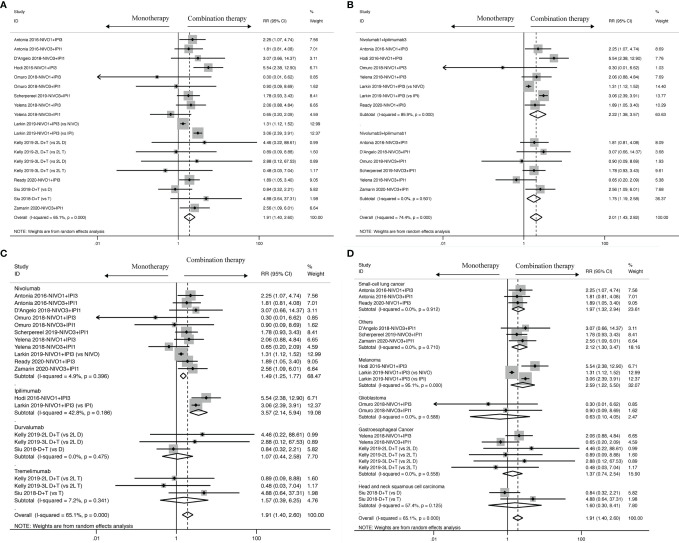
ORR analysis of RR and 95% CI for cancer patients treated with ICI-based combination therapy *versus* monotherapy in all included studies **(A)**, and on the basis of specific dosage **(B)**, control drugs **(C)** and tumor types **(D)**.

Nine studies contain the data of objective response used to calculate the ORR. The results revealed that ICI-based combination therapy had an ORR benefit compared to ICI-based monotherapy (RR: 1.91, 95% CI: 1.40 to 2.60) as displayed in **Figure 5**, and moderate heterogeneity was identified in ORR analysis (I^2^ = 65.1%, *P < 0.001*). Ergo, we found that similar ORR was reported for Nivolumab1 + Ipilimumab3 (RR: 2.22, 95% CI: 1.38 to 3.57) and Nivolumab3 + Ipilimumab1 (RR: 1.75, 95% CI: 1.19 to 2.58) with statistical significance. Moreover, the heterogeneity reduced from moderate to none in specific dosage of Nivolumab3 + Ipilimumab1 (I^2^ = 0.0%, *P = 0.501*), whereas higher heterogeneity was observed in Nivolumab1 + Ipilimumab3 (I^2^ = 85.9%, *P = 0.000*).

Meanwhile, in terms of drug control, better ORR was found in Nivolumab (RR: 1.49; 95% CI: 1.25 to 1.77) and Ipilimumab (RR: 3.57; 95% CI: 2.14 to 5.94) rather than in Durvalumab (RR:1.07; 95% CI: 0.44 to 2.58) and Tremelimumab (RR: 1.57; 95% CI: 0.39 to 6.25). In Nivolumab (I^2^ = 4.9%, *P = 0.396*), Durvalumab (I^2^ = 0.0%, *P = 0.475*), Tremelimumab (I^2^ = 7.2%, *P = 0.341*), heterogeneity was observed to sharply decline, but in Ipilimumab, it just diminished from moderate to mild (I^2^ = 42.8%, *P = 0.186*).

In addition to dosage in the intervention group and drug control, it was observed that tumor type also profoundly influences objective response in small-cell lung cancer (RR: 1.97, 95% CI: 1.32 to 2.94) and melanoma (RR:2.59, 95% CI: 1.22 to 5.50), but glioblastoma (RR: 0.63, 95% CI: 0.10 to 4.05) and gastroesophageal cancer (HR: 1.37, 95% CI: 0.74 to 2.54) didn’t get crucial improvement. Small-cell lung cancer (I^2^ = 0.0%, *P = 0.912*), glioblastoma (I^2^ = 0.0%, *P = 0.588*) and gastroesophageal cancer (I^2^ = 0.0%, *P = 0.558*), other than melanoma (I^2^ = 95.1%, *P < 0.001*), were associated with a decrease of heterogeneity.

### Safety

ICIs could easily trigger off some common all-grade and high-grade immune-related AEs, as shown in **Table 2**. Patients treated with ICI-based combination therapy suffered an increased all-grade risk of IRAEs, including rash (*P < 0.001*), pruritus (*P < 0.001*), diarrhea (*P < 0.001*), nausea (*P < 0.001*), vomiting (*P < 0.001*), fatigue (*P < 0.001*), hypopituitarism (*P < 0.001*), pneumonitis (*P < 0.001*), ALT increased (*P < 0.001*), AST increased (*P < 0.001*), and appetite decreased (*P < 0.001*).

**Table 2:** Treatment-related common adverse events in this meta-analysis.

Adverse events	RR (95% CI)			
	All Grade	*P* value	Grade≥3	*P* value
Rash	1.52 (1.30, 1.77)	<0.001	2.83 (1.48, 5.44)	0.002
Pruritus	1.28 (1.12, 1.46)	<0.001	4.69 (1.50, 14.63)	0.008
Diarrhea	1.70 (1.50, 1.92)	<0.001	2.23 (1.60, 3.10)	<0.001
Nausea	1.64 (1.37, 1.97)	<0.001	2.47 (1.28, 4.78)	0.007
Vomiting	2.47 (1.28, 4.78)	0.007	2.14 (1.04, 4.42)	0.040
Hypopituitarism	2.12 (1.67, 2.70)	<0.001	3.53 (0.74, 16.87)	0.110
Pneumonitis	2.84 (1.80, 4.48)	<0.001	1.84 (0.80, 4.26)	0.150
Fatigue	1.20 (1.08, 1.33)	<0.001	1.97 (1.24, 3.12)	0.004
ALT increased	3.34 (2.51, 4.44)	<0.001	4.56 (2.79, 7.46)	<0.001
AST increased	2.79 (2.19, 3.57)	<0.001	4.25 (2.61, 6.91)	<0.001
Decreased appetite	1.42 (1.17, 1.72)	<0.001	1.63 (0.77, 3.44)	0.200

Moreover, we further calculated the risk of high-grade AEs to evaluate the severity among multiple AEs. Similar to all-grade AEs, cancer patients who received ICI-based combination therapy also failed to report a lower risk of high-grade AEs, and rash (*P = 0.002*), pruritus (*P = 0.008*), diarrhea (*P < 0.001*), nausea (*P = 0.007*), vomiting (*P = 0.040*), fatigue (*P = 0.004*), ALT increased (*P < 0.001*) and AST increased (*P < 0.001*) all received a higher risk of high-grade AEs. Furthermore, pneumonitis (*P = 0.150*), hypopituitarism (*P = 0.110*), and decreased appetite (*P = 0.200*) showed a trend towards increased risk in patients treated with ICI-based combination therapy as compared with monotherapy but did not differ significantly.

### Sensitivity Analysis and Publication Bias

Sensitivity analysis was performed to estimate the possible effect of single-study results in accordance to pooled OS (**Supplementary Figure 4**). Judging from the analysis, the pooled outcome was not notably influenced by single-study removal, which demonstrated the reliability and rationality of this meta-analysis.

**Figure 6:**
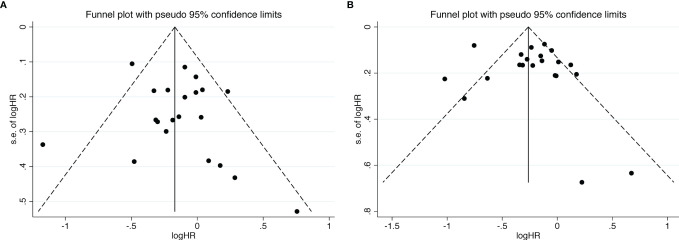
Funnel plots of OS **(A)** and PFS **(B)** from the included studies for the visual detection of systematic publication bias and small study effect.

We further used funnel plot to explore the existence of publication bias (**Figure 6**). All studies included slightly exhibited asymmetric distribution on the funnel plots of OS and PFS, which revealed that the potential publication bias should not be discounted across all studies. Meanwhile, Egger’s test was used to examine the publication bias on the basis of OS and PFS, and the results didn’t significantly identified overall risk of publication bias (OS: *P = 0.371*; PFS: *P = 0.531*) (**Supplementary Figure 5**). Due to its reliability and sensitivity, Begg’s test was also performed in our meta-analysis when there were more than twenty included effect sizes (OS: *P = 0.904*; PFS: *P = 0.672*) (**Supplementary Figure 6**).

## Discussion

ICI-based combination therapy is the first therapeutic option for cancer patients. After analyzing the thirteen included studies, the pooled results indicated that ICI-based combination therapy was superior to monotherapy, whether in OS, PFS, or ORR. However, there were still some safety issues that were badly in need of effective management. Why is combination therapy more efficient than monotherapy as a treatment for multiple cancers? Firstly, in terms of the fact that different ICIs are based on varied mechanisms of ‘time and space’, the combination therapy of anti-CTLA-4 and anti-PD-1/PD-L1 was supposed to activate the antitumor immune response synergistically to enhance the response rate (35, 36). Secondly, combination therapy promotes the recruitment and activation of T cells, which induce the expression of CTLA-4 and PD- 1/PD-L1 (37–39). Thirdly, two ICIs in combination were able to aggravate the production of inflammatory cytokines and further stimulate the immune response (40, 41). Last but not least, combination use of two ICIs results in a higher ratio of effector to regulatory T cells, which causes antagonism of pro-anergeric signaling through Tregs (42–44).

At present, despite the fact that there is no longer a compulsive requirement to detect PD-L1 expression in some indications approved by the FDA, PD-L1 expression must be taken into consideration in the clinical application of ICIs. The pooled outcomes of our meta-analysis differ from the previous view that cancer patients with positive PD-L1 expression failed to benefit from two ICIs in combination, but those with negative PD-L1 did, whether in OS or PFS analysis. Differently, the efficacy of combination therapy is limited by low response rates of any PD-L1 expression, with only a minority of patients responding to it. Therefore, PD-L1 expression is likely to be considered as biomarker to evaluate the survival time or prognosis of cancer patients treated with ICIs, but it is not available to predict the tumor progression. Furthermore, these results revealed that monotherapy of ICIs remains the preferred treatment option compared with combination therapy of two ICIs for cancer patients with positive PD-L1 expression. Meanwhile, cancer patients with less PD-L1 expression benefit more from therapies combining anti-PD-1/PD-L1 and anti-CTLA-4, which possibly results from synergistic activation of antitumor immune response. However, it’s a pity that we only chose the cut-off value 1% to explore the effects of PD-L1 expression and thus failed to get valid data to further analyze the important role of PD-L1 expression in two ICIs in combination response. The reason is that only a small sample size of enrolled studies explored the PD-L1 proportion score with 10and 50% cut-off values in terms of OS and PFS, which was underpowered for statistical analysis on survival. Meanwhile, <1% confirmed to have a better clinical value because we found <1% was the most widely used PD-L1 expression in clinical research. However, we’re interested in the interaction on the basis of multiple cut-off values and will attempt to make further study. In general, it is not necessary to detect the PD-L1 expression before receiving two ICIs in combination, because even though PD-L1 is positively expressed in tumor and immune cells, there’s still no obvious survival benefit to cancer patients.

In addition to PD-L1 expression, different dosages also have direct influence on the efficacy of ICI-based combination therapy. Although OS benefit was only observed in the ‘Nivolumab1 + Ipilimumab3’ subset and was not found in the ‘Nivolumab3 + Ipilimumab1’ subset in our meta-analysis, the FDA has approved high-dosage Nivolumab combined with low-dosage Ipilimumab as optimal dosage for ICI-based combination therapy since 2018, which probably results from the fact that CLTA-4 distributes on T cells, and its receptor is unable to specifically expressed in tumor tissues (45). Thus, it’s easy to activate the whole immune system and anti-CLTA-4 inhibitors, which tends to increase the incidence of adverse reactions and lower the quality of life (46). Those adjustments of dosage increase tolerance to ICIs and guarantee its efficacy at the same time, making it possible in clinical practice. Based on the fact that OS benefit favored combination therapy in patients compared with Ipilimumab in terms of control drugs and the result from subgroup analysis based on specific target of control drugs, we can draw a conclusion that anti-PD-1/PD-L1 inhibitors have an OS advantage over anti-CTLA-4 inhibitors (47, 48), and it’s unnecessary for cancer patients to receive combination therapy of two ICIs after anti-PD-1/PD-L1 inhibitors for more long-term benefit. But those who have taken anti-CTLA-4 inhibitors could consider it. Besides, a sharp improvement was observed when compared with Nivolumab and Ipilimumab in PFS analysis. Although PFS survival benefit was not observed from Durvalumab, when taking the outcome from subgroup analysis based on specific target of control drugs into consideration, anti-PD-L1 inhibitors were related to better PFS benefit as monotherapy, and cancer patients could replace anti-PD-1 and anti-CTLA-4 inhibitors with two ICIs in combination. Beyond that, better objective response was reported in melanoma (49) and small-cell lung cancer (50), which could be related to higher tumor mutation burden in those tumor types. While others said the efficacy of ICI-based monotherapy or combination therapy is irrelevant to tumor types, so far, specific reasons have remained unknown.

According to the searches resulting from Clinical Trials, a total of 85 running or pending clinical trials in relation to ICI-based combination therapy have been registered to study the efficacy, and some trials have tested the safety of the treatment for cancer while others haven’t. Among those trials, the majority performed with Nivolumab and Ipilimumab reached about 70%, while the trials performed with Durvalumab and Tremelimumab reached only 30%. The number of registered clinical trial participants ranges from five to 1,360, including 0–49 persons enrolled in 36 trials, 50–99 persons in 19 trials, 100–199 persons in 14 trials, 200–499 persons in 10 trials, and above 1,000 persons in two trials. According to cancer type, melanoma dominates the highest part (15 trials), followed by lung cancer (11 trials, including non-small cell lung cancer and small cell lung cancer), renal cell carcinoma (eight trials), colorectal cancer (five trials), breast cancer (four trials), and so on.

Nivolumab plus Ipilimumab, as an immune treatment combination, has been used to cure wild-type BRAF V600 melanoma since it was first approved in September 2015 by the FDA. Its clinical indication was extended to metastatic melanoma in January 2016, for advanced renal cell carcinoma as first-line treatment in April 2018, and for metastatic colon cancer patients after using Fluorouracil, Oxaliplatin, and Irinotecan with MSH-H and dMMR in July 2018, for hepatocellular carcinoma after being treated with Sorafenib (49). Moreover, Bristol-Myers Squibb announced that the FDA accepted their supplemental Biologics License Application of Nivolumab and Ipilimumab plus finite-duration chemotherapy for metastatic and recurrent non-small cell lung cancer without ALK fusion, and EGFR mutation as first-line treatment in April 2020. Several days later, this company reported that Check Mate-743, the key phase III clinical trial, reached the primary endpoint with OS improvement of clinical and statistical significance in previously untreated malignant pleural mesothelioma, and thus held the promise of submitting an application to the FDA. However, no guideline recommendation of ICI-based combination for cancer treatment has been published.

Recently, ICI-based combination therapy brings a new hope to cancer treatment. Its efficacy has initially been proved to be reliable as well as advantageous, and some of the steps have been taken to decrease the side effects. This meta-analysis provides us with valuable insights as we look to the future of ICI-based combination. Firstly, most of two ICIs in combination have been approved by the FDA as subsequent-line treatments for patients with multiple cancers, and thus more clinical trials are badly needed with the aim of pushing the indications to first-line therapy. It is urgent to confirm its clinical value to cancer patients, rather than an alternative scheme. Secondly, the specific medication regimen with optimal efficacy of ICI-based combination therapy, including specific drug-pair, dosage, order, frequency, duration and reasonable choice drug time, remains unknown. So, it’s essential to perform the dose-escalation test on the basis of previous study data after confirming certain combination therapies are rational and scientifically explored in animal models and then to explore the specific dosage across the whole clinical trial progress and make further improvement and development after it becomes available on the market. Besides, except for ICIs, it is also far from clear how to screen the benefit for the population and develop the personalized treatment. Although studies have showed that tumor mutation burden (TMB), a measure of the number of somatic protein-coding base substitution and insertion or deletion mutations occurring in a tumor specimen, is relevant with the efficacy and response of ICIs (51), which may serve as a biomarker to predict prognosis, TMB still fails to be a principal biomarker, owing in part to lack of standardization and difficulty in pathologist interpretation of immune cell state (52, 53). Hence, these clinical uses are urgently in need of exploring the features of immunophenotype and screening out the biomarkers of changes to the immune microenvironment. Fourthly, although the studies on two ICIs in combination are relatively mature, it is still advisable to combine ICI with adoptive cell therapy, cytokine therapy, immune agonist, oncolytic virus, vaccine, and other immune therapies in order to improve the curative effect. Moreover, all relevant trials of ICI-based combination therapy were already under way, whereas small samples were employed in most of the clinical trials, and each cancer’s response duration differed from others. Therefore, more prospective clinical trials, or RCTs with rigorous design and large samples are urgently needed to provide reliable evidence for cancer treatment. Finally, for expected therapeutic efficacy, ICI-based combination therapy should lower the dosage to the minimum to reduce and even prevent its adverse reactions. Despite the fact that two ICIs in combination have achieved satisfactory effect, attention should also be paid to potential toxicity, especially the IRAEs. Although prior studies have found that it is expected to manage the side effects of combination therapy, merely a few serious adverse events are reported (14). Therefore, it’s crucial to optimize the management of adverse events for timely access to early prevention, correct recognition, and proper treatment.

The empirical results reported herein should take some limitations into consideration. First of all, the data in our meta-analysis were on the basis of study-level evidence instead of individual results, which may lower the reliability by ignoring some extreme values. Secondly, we failed to explore the potential factors that may influence the response to ICI-based combination therapy and ended up with heterogeneity because only a few included studies provided the data to perform subgroup or meta-regression analysis. Thirdly, given the fact that the analysis was based on the number of participants since most of the included studies were performed with small sample, it is hard to draw exact results for this potential bias. Fourthly, some definitions of adverse events were not uniform. For example, some studies reported diarrhea and colitis individually, while others reported their compilations. These possible repeat cases lead to the result that we were unable to collect the data from ‘diarrhea’, ‘colitis’, and ‘diarrhea or colitis’ at the same time. Furthermore, we did not analyze the efficacy and safety in terms of PD-L1 expression or other biomarkers to identify the specific population with the most survival benefit.

## Conclusion

Broadly speaking, we performed a direct comparison and verified that ICI-based combination therapy offers a better survival benefit than monotherapy. Besides, combination therapy was the optimum treatment for cancer, especially when using specific dosage and regimen to treat melanoma and small-cell lung cancer with no absolute demand for the detection of PD-L1 expression. Meanwhile, potential toxicity should also be taken into consideration seriously, especially the IRAEs. Further studies on this promising immunotherapeutic approach not only remain to enhance the long-term efficacy and minimize the adverse reaction, but also explore potential biomarkers, suitable indications and optimal patients to acquire the most benefit from ICI-based combination therapy.

## Data Availability Statement

The original contributions presented in the study are included in the article/**Supplementary Material**. Further inquiries can be directed to the corresponding author.

## Author Contributions

LZ and LS contributed equally to this work. SR and HW contributed to the design and conception of the study. JY, YZ, and YL carried out the collection and assembly of data. LZ and LS performed the data analysis, interpretation, and statistical analysis. LZ and LS wrote the manuscript. MS revised the manuscript, and then SR gave the final approval of the manuscript. All authors contributed to the article and approved the submitted version.

## Funding

This study was financially supported by the Natural Science Foundation of Zhejiang Province (SR, no. LY17H270007); National Natural Science Foundation of China (MS, no. 81573902); General Research Program for Education of Zhejiang Provincial (LS, No. Y202045212); Cultivation Program for Innovative Talent Graduate Students (LZ; No. 721100G00713); China Postdoctoral Science Foundation (SR, no. 2017M612040/2018T110610); Zhejiang Provincial Program for the Cultivation of High-Level Innovative Health Talents (SR, no. 2015-43); Program for the Cultivation of Youth talents in China Association of Chinese Medicine (SR, no. QNRC2-C08); Zhejiang Provincial Program for the Cultivation of the Young and Middle-Aged Academic Leaders in Colleges and Universities (SR, no. 2017-248); and Zhejiang Provincial Project for the key discipline of Traditional Chinese Medicine (Yong Guo, no. 2017-XK-A09).

## Conflict of Interest

The authors declare that the research was conducted in the absence of any commercial or financial relationships that could be construed as a potential conflict of interest.
