# Supplementary material for: Association of Survival and Immune-Related Adverse Events With Anti-PD-1/PD-L1 and Anti-CTLA-4 Inhibitors, Alone or Their Combination for the Treatment of Cancer: A Systematic Review and Meta-Analysis of 13 Clinical Trials
Source: Front Oncol. 2021 Feb 25;11:575457. doi: 10.3389/fonc.2021.575457 (PMC7947606; doi:10.3389/fonc.2021.575457)
Supplement: Supplementary file 1 [file DataSheet_1.docx]

**SUPPLEMENTARY MATERIAL**

**Supplementary Method**

**Supplementary Method 1 -** Search strategy for PubMed

**Supplementary Table**

**Supplementary Table 1 -** Further Characteristics of the included trial

**Supplementary Figure**

**Supplementary Figure 1 -** Risk of bias graph

**Supplementary Figure 2 -** Risk of bias summary

**Supplementary Figure 3 -** PD-L1 biomarker analysis of ORR

**Supplementary Figure 4 -** Sensitivity analysis of OS and PFS

**Supplementary Figure 5 -** Egger’s test of OS and PFS

**Supplementary Figure 6 -** Begg’s test of OS and PFS

**Supplementary Method 1**– Search strategy for PubMed

((((((Neoplasms[MeSH Terms]) OR Neoplasm*[Title/Abstract]) OR tumor*[Title/Abstract]) OR Cancer*[Title/Abstract]) OR malignan*[Title/Abstract]) OR carcinoma*[Title/Abstract]) OR tumour*[Title/Abstract]) OR adenocarcin*[Title/Abstract])))) AND ((((((((((((((((((((((((((((((((Immunosuppressive Agents[MeSH Terms]) OR Immunosuppress*[Title/Abstract]) OR immune checkpoint inhibitors[Title/Abstract]) OR ICIs[Title/Abstract]) OR Programmed Death 1[Title/Abstract]) OR Programmed Death Ligand 1[Title/Abstract]) OR Cytotoxic T Lymphocyte Antigen 4) OR PD-1[Title/Abstract]) OR PD1[Title/Abstract]) OR PD-L1[Title/Abstract]) OR PDL1[Title/Abstract]) OR CTLA-4[Title/Abstract]) OR CTLA4[Title/Abstract]) OR Ipilimumab[Title/Abstract]) OR Yervoy[Title/Abstract]) OR Tremelimumab[Title/Abstract]) OR Atezolizumab[Title/Abstract]) OR Tecentriq[Title/Abstract]) OR Durvalumab[Title/Abstract]) OR Imfinzi[Title/Abstract]) OR Pembrolizumab[Title/Abstract]) OR lambrolizumab[Title/Abstract]) OR Keytruda[Title/Abstract]) OR Nivolumab[Title/Abstract]) OR Opdivo[Title/Abstract]) OR Avelumab[Title/Abstract]) OR Bavencio[Title/Abstract]) OR Cemiplimab[Title/Abstract]) OR Camrelizumab[Title/Abstract]) OR Toripalimab[Title/Abstract]) OR Sintilimab[Title/Abstract]) OR Libtayo[Title/Abstract])) AND (((((single[Title/Abstract]) OR alone[Title/Abstract]) OR combination[Title/Abstract]) OR combined with[Title/Abstract]) OR plus[Title/Abstract])) AND ((((((((((randomized controlled trial[Publication Type]) OR controlled clinical trial[Publication Type]) OR randomized[Title/Abstract]) OR placebo[Title/Abstract]) OR randomly[Title/Abstract]) OR "Clinical Trials as Topic"[Mesh:NoExp]) OR trial[Title])) NOT ((((animals[MeSH Terms]) NOT ((humans[MeSH Terms]) AND animals[MeSH Terms])))))))))

**Supplementary Table 1**- Further Characteristics of the included trial

| Author, year | Follow-up, months | Intervention arm | | Control arm | |
| --- | --- | --- | --- | --- | --- |
|  |  | Regimen | Median age, years | Regimen | Median age, years |
| Antonia, 2016 | 12.0 vs 3.3 | Nivolumab+Ipilimumab | 66.0 (58 - 71) | Nivolumab | 63.0 (57 - 68) |
|  | 8.7 vs 3.3 |  | 61.0 (56 - 65) |  |  |
| D'Angelo, 2018 | 14.2 vs 13.6 | Nivolumab+Ipilimumab | 57.0 (27 - 81) | Nivolumab | 56.0 (21 - 76) |
| Hodi, 2016 | 24.5 | Nivolumab+Ipilimumab | 64.0 (27 - 87) | Ipilimumab | 67.0 (31 - 80) |
| Long, 2018 | 14.0 vs 17.0 | Nivolumab+Ipilimumab | 59.0 (53 - 68) | Nivolumab | 63.0 (52 - 74) |
| Omuro, 2018 | 24.0 vs 28.0 | Nivolumab+Ipilimumab | 57.0 (37 - 68) | Nivolumab | 58.5 (42 - 73) |
|  | 22.0 vs 28.0 |  | 60.0 (27 - 73) |  |  |
| Scherpereel, 2019 | 20.1 | Nivolumab+Ipilimumab | NA | Nivolumab | NA |
| Yelena, 2018 | 24.0 vs 28.0 | Nivolumab+Ipilimumab | 53.0 (27 - 77) | Nivolumab | 60.0 (29 - 80) |
|  | 22.0 vs 28.0 |  | 58.0 (19 - 81) |  |  |
| Larkin, 2019 | 54.6 vs 36.0 | Nivolumab+Ipilimumab | 61.0 (18 - 88) | Nivolumab | 60.0 (25 - 90) |
|  | 54.6 vs 18.6 |  |  | Ipilimumab | 62.0 (18 - 89) |
| Kelly, 2019 | 9.2 vs 3.5 | Durvalumab+Tremelimumab (2L) | 64.0 (27 - 78) | Durvalumab | 60.0 (29 - 79) |
|  | 9.2 vs 9.2 |  |  |  |  |
|  | 10.7 vs 3.5 | Durvalumab+Tremelimumab (3L) | 59.0 (35 - 75) | Tremelimumab | 54.0 (19 - 74) |
|  | 10.7 vs 9.2 |  |  |  |  |
| O'Reilly, 2019 | 3.2 | Durvalumab+Tremelimumab | NA | Durvalumab | NA |
| Ready, 2020 | 11.2 vs 11.9 | Nivolumab+Ipilimumab | 63.0 (29 - 83) | Nivolumab | 65.0 (41 - 91) |
| Siu, 2018 | 6.5 vs 6.0 | Durvalumab+Tremelimumab | 62.0 (26 - 81) | Durvalumab | 62.0 (23 - 82) |
|  | 6.5 vs 5.2 |  |  | Tremelimumab | 61.0 (42 - 77) |
| Zamarin, 2020 | 33.5 vs 33.1 (Frist-stage) | Nivolumab+Ipilimumab | 62.0 (38 - 92) | Nivolumab | 63.0 (37 - 87) |
|  | 11.0 vs 10.7 (Second-stage) |  |  |  |  |

**Abbreviations:** NA, not available.

**Supplementary Figure 1**- Risk of bias graph: Review authors' judgments about each risk of bias item presented as percentages across all included studies.


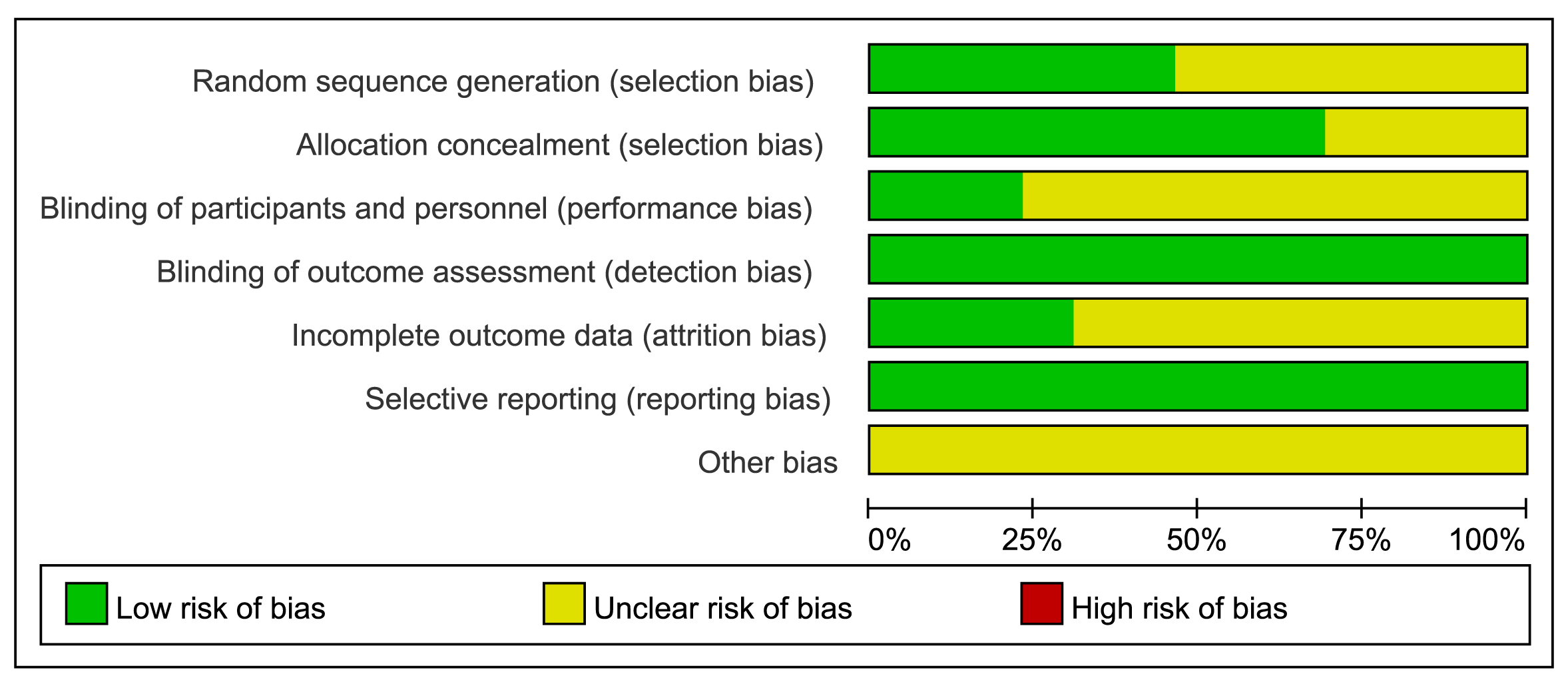


**Supplementary Figure 2**- Risk of bias summary: Review authors’ judgments about each risk of bias item for each included study.


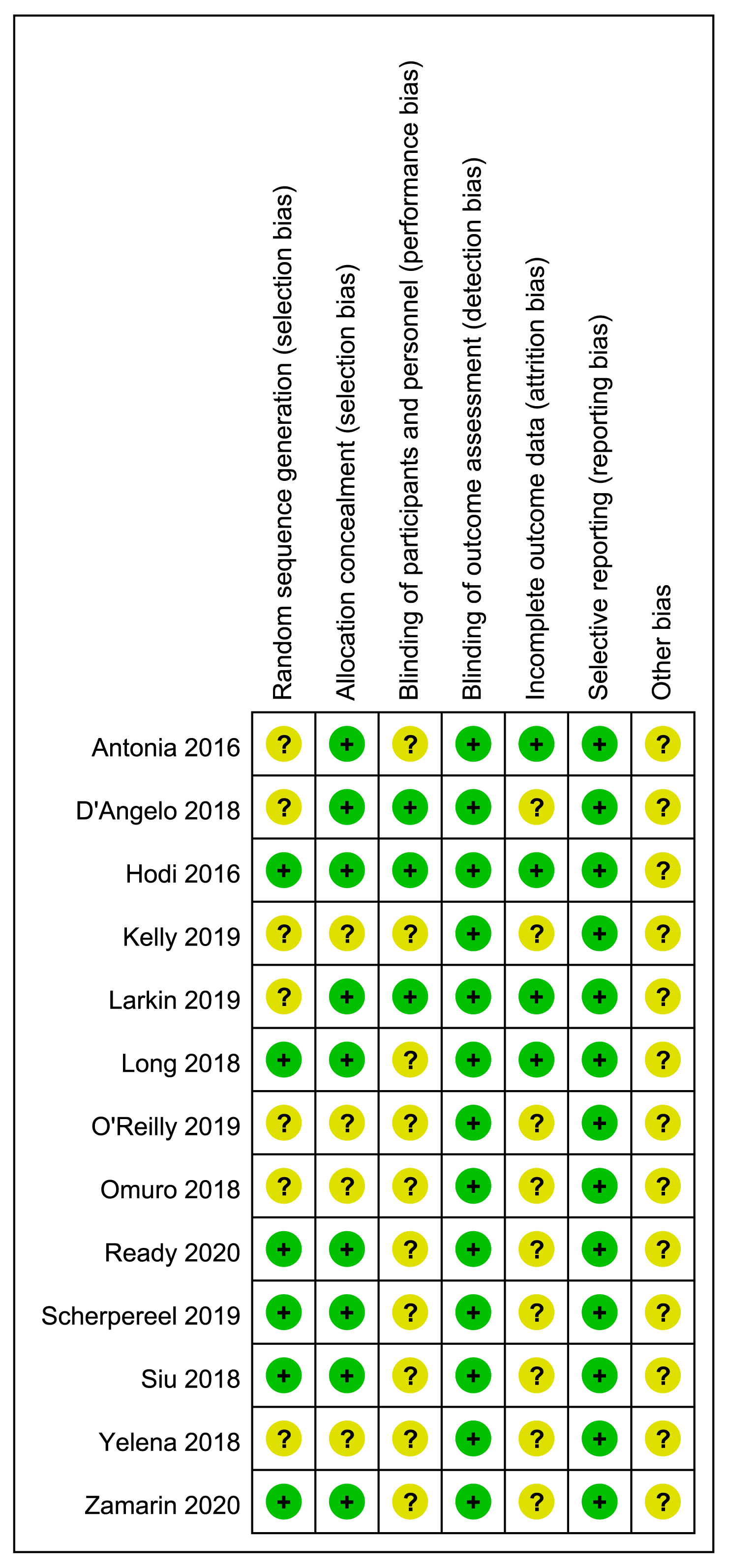


**Supplementary Figure 3**- PD-L1 biomarker analysis: Analysis of PD-L1 expression pooled HR and 95%CI of ORR for cancer patients assigned to combination therapy, compared with those assigned to monotherapy.

**Supplementary Figure 4**- Sensitivity analysis: Sensitivity analysis of OS (A) and PFS (B) in included studies for the robustness of findings to different aspects of the trials methodology.

**A**

**
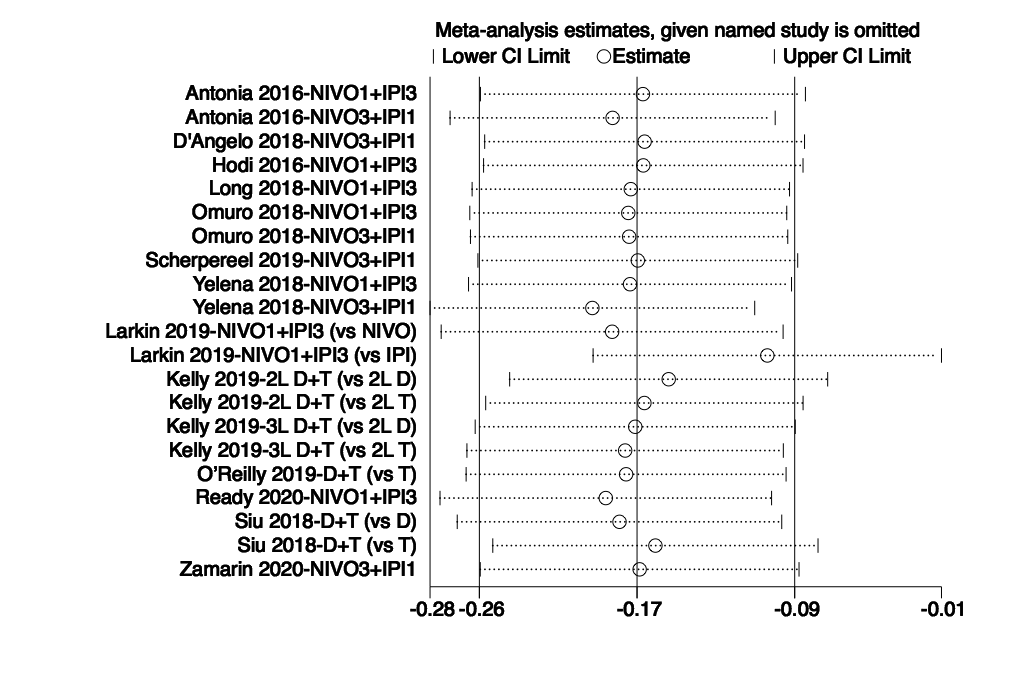
**

**B
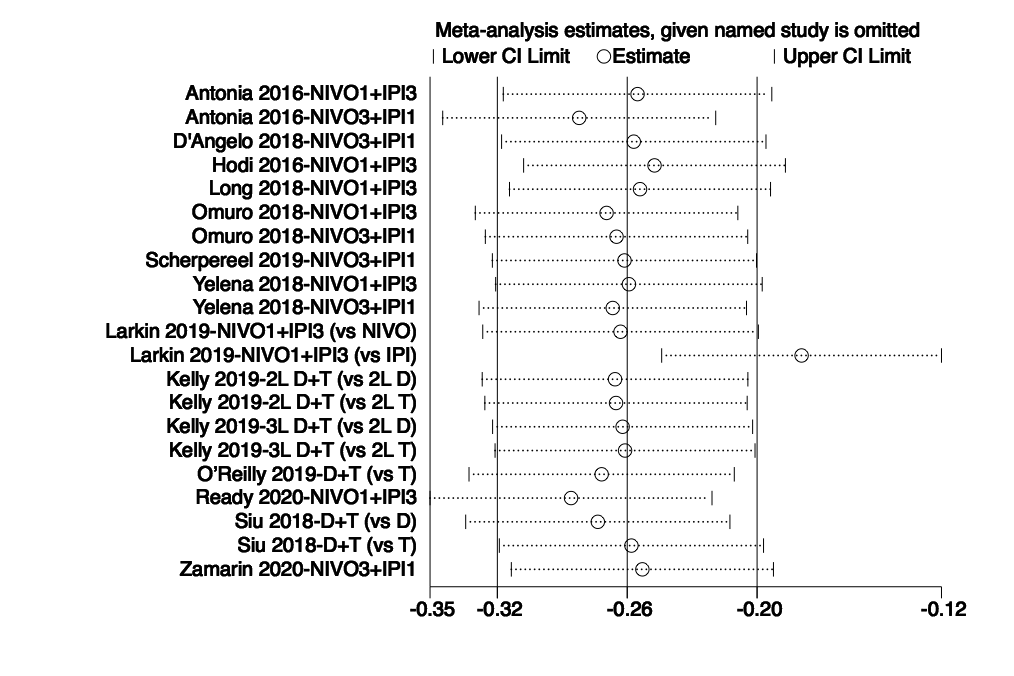
**

**Supplementary Figure 5**- Egger’s test: Egger’s test of OS (A) and PFS (B) from in included RCTs for calculative detection of association between the study effects and the study size.

**A**

**
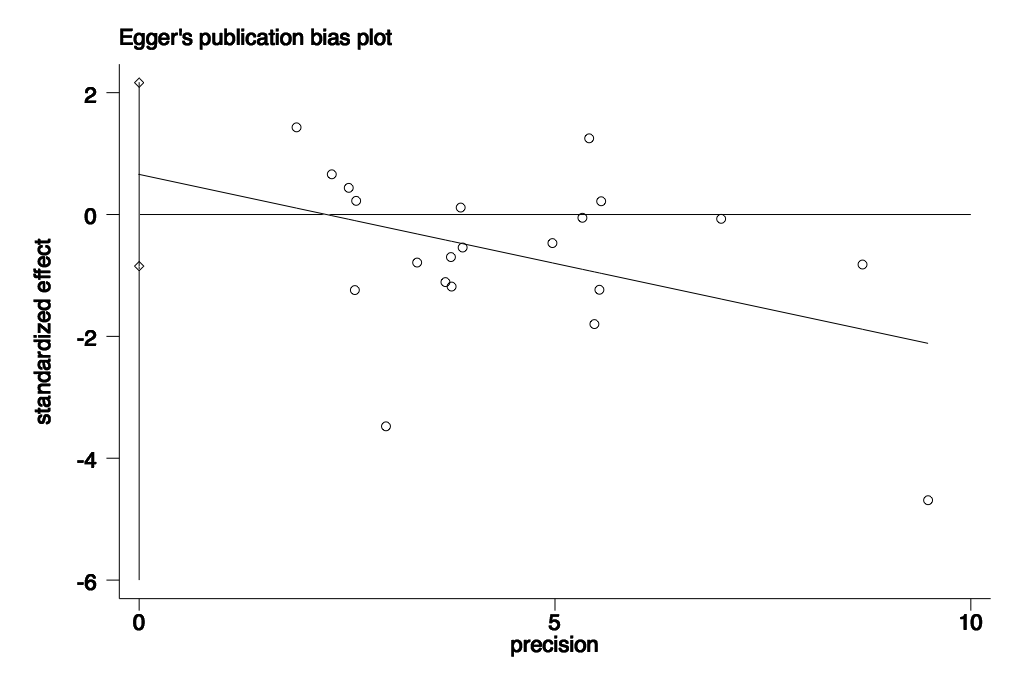
**

**B**

**
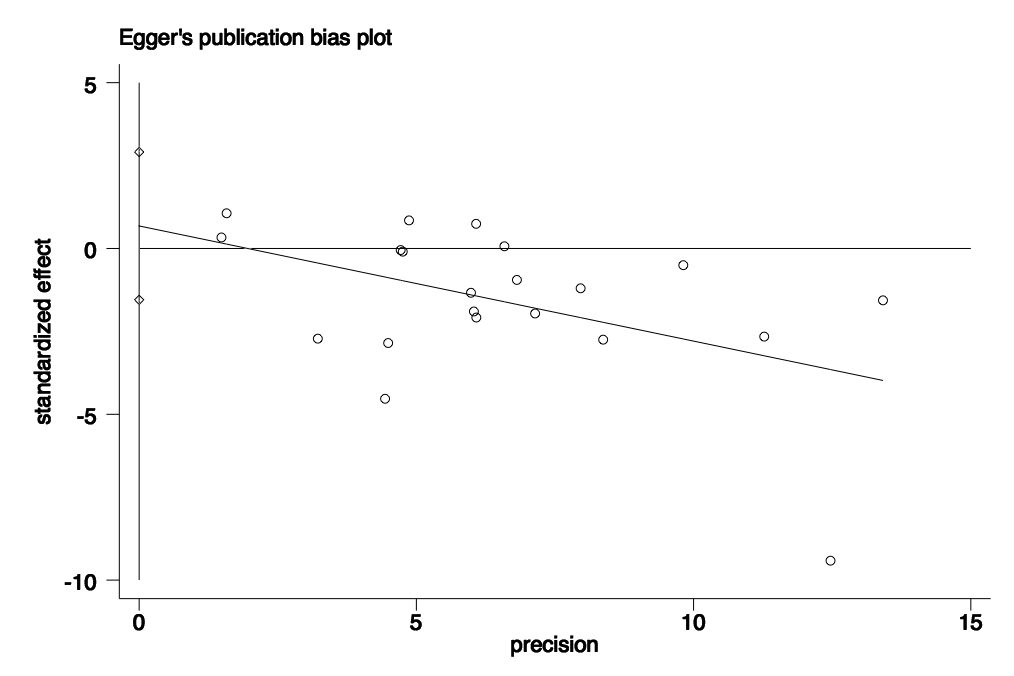
**

**Supplementary Figure 6**- Begg’s test: Begg’s test of OS (A) and PFS (B) from in included RCTs for calculative detection of association between the study effects and the study size.

**A**

**
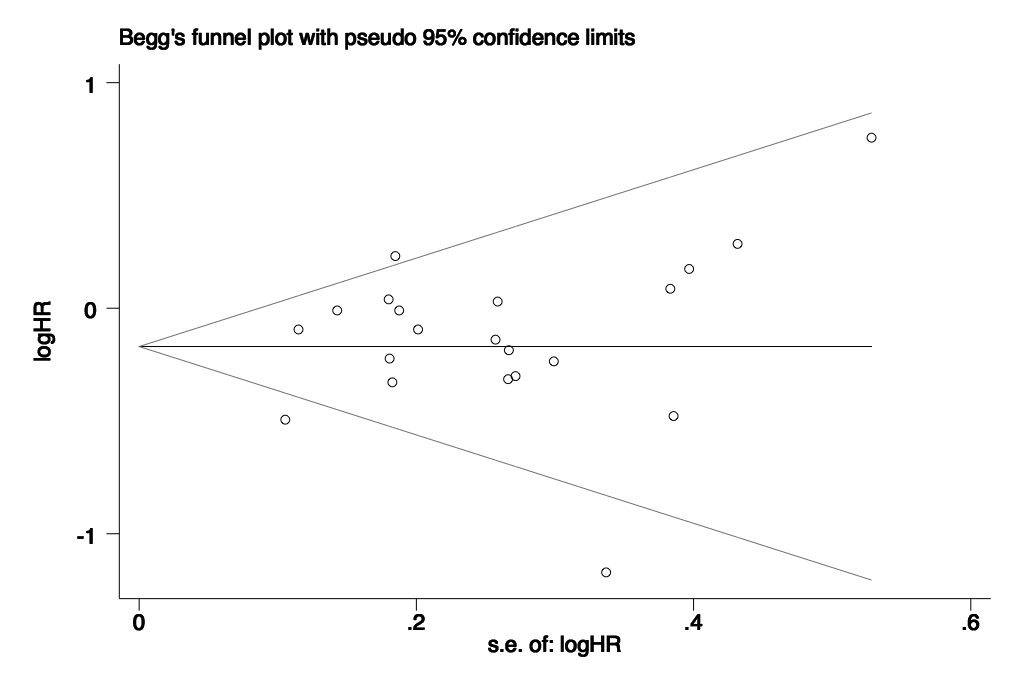
**

**B**

**
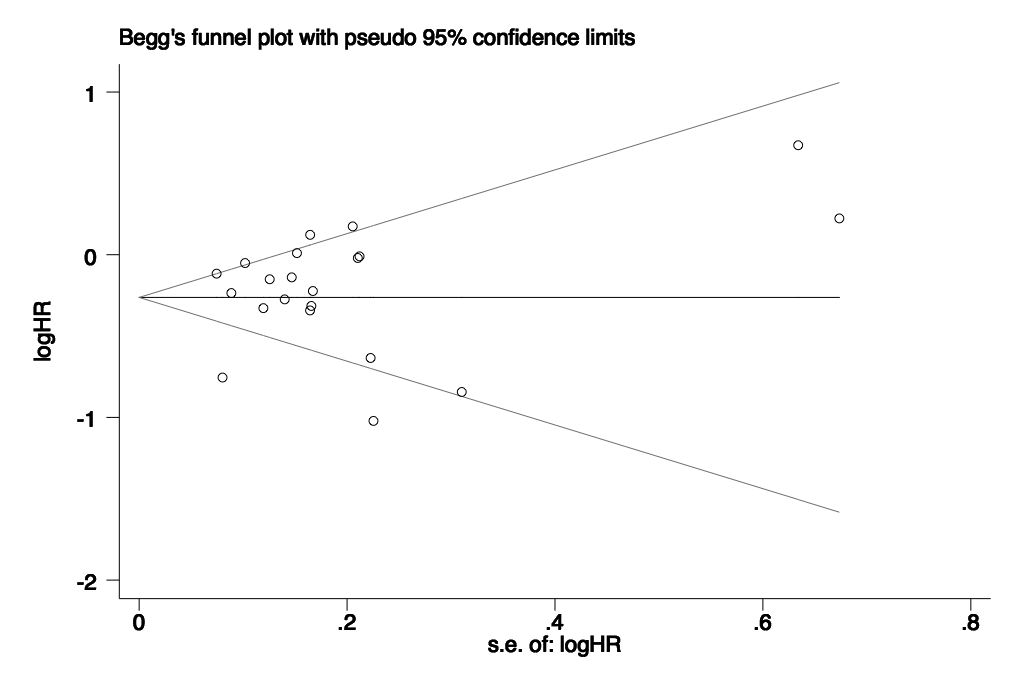
**
